# Direct Growth of CuO Nanorods on Graphitic Carbon Nitride with Synergistic Effect on Thermal Decomposition of Ammonium Perchlorate

**DOI:** 10.3390/ma10050484

**Published:** 2017-05-02

**Authors:** Linghua Tan, Jianhua Xu, Shiying Li, Dongnan Li, Yuming Dai, Bo Kou, Yu Chen

**Affiliations:** 1 National Special Superfine Power Engineering Research Center, Nanjing University of Science and Technology, Nanjing 210094, China; joshldn@163.com; 2 School of Chemical Engineering, Nanjing University of Science and Technology, Nanjing 210094, China; xujianhua931118@163.com (J.X.); lishiying0613@163.com (S.L.); 3 Jiangsu Key Laboratory of Advanced Structural Materials and Application Technology, School of Materials Engineering, Nanjing Institute of Technology, Nanjing 211167, China; dym@njit.edu.cn (Y.D.); koutianbo@hotmail.com (B.K.); cy_5432@163.com (Y.C.)

**Keywords:** g-C_3_N_4_, CuO, Nanocomposite, catalysis, synergistic effect

## Abstract

Novel graphitic carbon nitride/CuO (g-C_3_N_4_/CuO) nanocomposite was synthesized through a facile precipitation method. Due to the strong ion-dipole interaction between copper ions and nitrogen atoms of g-C_3_N_4_, CuO nanorods (length 200–300 nm, diameter 5–10 nm) were directly grown on g-C_3_N_4_, forming a g-C_3_N_4_/CuO nanocomposite, which was confirmed via X-ray diffraction (XRD), transmission electron microscopy (TEM), field emission scanning electron microscopy (FESEM), and X-ray photoelectron spectroscopy (XPS). Finally, thermal decomposition of ammonium perchlorate (AP) in the absence and presence of the prepared g-C_3_N_4_/CuO nanocomposite was examined by differential thermal analysis (DTA), and thermal gravimetric analysis (TGA). The g-C_3_N_4_/CuO nanocomposite showed promising catalytic effects for the thermal decomposition of AP. Upon addition of 2 wt % nanocomposite with the best catalytic performance (g-C_3_N_4_/20 wt % CuO), the decomposition temperature of AP was decreased by up to 105.5 °C and only one decomposition step was found instead of the two steps commonly reported in other examples, demonstrating the synergistic catalytic activity of the as-synthesized nanocomposite. This study demonstrated a successful example regarding the direct growth of metal oxide on g-C_3_N_4_ by ion-dipole interaction between metallic ions, and the lone pair electrons on nitrogen atoms, which could provide a novel strategy for the preparation of g-C_3_N_4_-based nanocomposite.

## 1. Introduction

Recently, graphitic carbon nitride (g-C_3_N_4_) has drawn considerable attention due to its unusual physical and chemical properties, which show great potential for the application in many aspects. g-C_3_N_4_ is metal-free, and has multiple structural defects, good thermal and chemical stability, and tunable electronic structure. More importantly, g-C_3_N_4_ is cost-effective and environmentally friendly. All these make g-C_3_N_4_ become a novel and promising form of catalysis and catalytic support [1,2,3,4,5,6,7,8]. To broaden the application of the g-C_3_N_4_-based nanocomposite, compounding functional nano-sized metal oxide materials with g-C_3_N_4_ has been of interest over recent years. The synergy of two functional materials will result in improved properties of the nanocomposite that will have a wider range of applications [9,10,11,12]. Specifically, Sridharan and his co-workers [13] reported the coupling of g-C_3_N_4_ with TiO_2_ by utilizing a thermal transformation methodology. With higher photocatalytic ability, the prepared composite greatly improved the degradation of methylene blue. Additionally, g-C_3_N_4_/CeO_2_ was prepared for the evaluation of its potential application in the hydrogen production by photocatalysis, and degradation of methylene blue [14,15]. g-C_3_N_4_/Bi_2_WO_6_ was also studied to explore its excellent properties [16,17,18,19,20]. For example, Li’s group [16] synthesized a series of g-C_3_N_4_/Bi_2_WO_6_ composites through a facile in situ hydrothermal approach, which demonstrated greatly enhanced responses to visible light. The selective reduction of CO_2_ to CO was remarkably enhanced. Analogously, Sierra et al [20] developed a Bi/Bi_2_WO_6_/g-C_3_N_4_ ternary composite via thermal reduction of Bi_2_WO_6_ with g-C_3_N_4_. Owing to its remarkable red-shift up to 620 nm, Bi/Bi_2_WO_6_/g-C_3_N_4_ could catalyze the degradation of methylene blue contaminants under visible light.

**Scheme 1:**
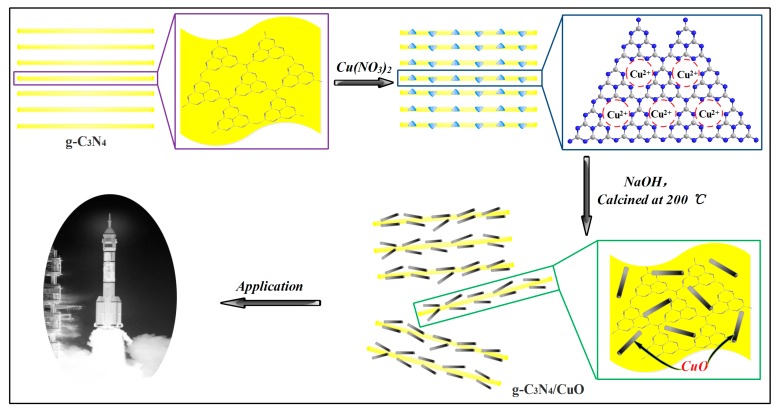
Illustration of the formation and application of the g-C_3_N_4_/CuO nanocomposite.

CuO is a typical p-type semiconductor that has been widely applied in photoelectric materials, gas sensors and catalysis [21,22]. The preparation of CuO nanostructures has received much attention in recent years [23,24]. Zhu et al [25] prepared a CuO:GO nanocomposite by a facile chemical method without the utilization of any templates or surfactants. Due to the good dispersion of CuO nanocrystals on GO nanosheets, the obtained CuO:GO nanocomposite showed a synergistic effect for the thermal decomposition of AP. Zabilskiy’s group [26] prepared a series of CuO crystals supported on CeO_2_ nanospheres using a glycothermal approach. The synthesized CuO/CeO_2_ exhibited very promising activity and stability, which could be effectively applied to the catalysis of N_2_O decomposition. Recently, Wang’s group reported on a new synthetic approach for the preparation of porous nanorod-type graphitic carbon nitride/CuO composites using a Cu-melamin supramolecular framework as a precursor [27]. These works have focused more on the control of the morphology of g-C_3_N_4_ than on that of CuO. Specifically, due to the three-dimensional hydrogen-bond (N—H⋯N) directions which guide the condensation of melamine molecules in the Cu-melamin precursor, the as-prepared CuO-g-C_3_N_4_ showed a porous nanorod morphology consisting of CuO nanoparticles spread on the surface of rod-shaped g-C_3_N_4_. However, the high reaction temperature (up to 550 °C) would lead to the serious sintering of CuO nanoparticles, which could affect the catalytic activity of the CuO-g-g-C_3_N_4_. Based on the above analysis, in order to obtain a novel g-C_3_N_4_/CuO nanocomposite with synergistic effect, the CuO nanoparticles were made to be uniformly dispersed on g-C_3_N_4_. Herein, we report on an improved strategy for the preparation of CuO nanorods well-dispersed g-C_3_N_4_ nanocomposite via the direct growth of CuO nanorods on g-C_3_N_4_, using a facile precipitation method (Scheme 1).

Ammonium perchlorate (AP), which accounts for 60% of the total mass of the propellant, is the most common oxidant in composite solid propellants [28]. The properties of solid propellants mostly depend on reaction rate, high-temperature decomposition, and the activation energy of AP thermal decomposition. Thus, exothermic heat and lower high-temperature decomposition (HTD) play an important role in the utilization of AP [29]. Up to now, as a transition metal oxide, CuO was demonstrated to be a promising catalyst for the thermal decomposition of AP [25]. Moreover, our group confirmed that g-C_3_N_4_ can also accelerate the thermal decomposition process [30]. It was anticipated that the direct growth of CuO on g-C_3_N_4_ will not only avoid the agglomeration of nano-sized CuO, but also efficiently accelerate the electron transfer rate between them. Thus, the thermal decomposition process of AP could be markedly accelerated in the presence of the novel g-C_3_N_4_/CuO catalyst.

## 2. Experimental

### 2.1. Materials

All the reagents were of analytical grade and used as received without further purification. Melamine, sodium hydroxide, cupric nitrate trihydrate and ethanol were all purchased from Sinopharm (Beijing, China). Distilled water was used throughout the experiment.

### 2.2. Preparation of g-g-C_3_N_4_/CuO Nanocomposite

Firstly, g-C_3_N_4_ solid was synthesized by heating melamine in a muffle furnace (Hefei Ke Jing Materials Technology Co., Ltd., Hefei, China) at 520 °C for two hours at a heating rate of 50 °C min^−1^ with air, the solid was then ground with agate mortar to obtain g-C_3_N_4_ powder. The product was washed with distilled water three times and dried at 70 °C [30].

Secondly, the g-C_3_N_4_/CuO nanocomposite was synthesized using a facile precipitation method [31]. The g-C_3_N_4_/CuO nanocomposite with differing CuO contents (5, 10, 20, 30, 50 wt %) were prepared and labeled as g-C_3_N_4_/*x* wt % CuO (*x* = 5, 10, 20, 30, 50). A typical experiment for the synthesis of g-C_3_N_4_/20 wt % CuO catalyst is as follows: 0.32 g of g-C_3_N_4_ was dispersed in 60 mL deionized water by ultrasonication for 30 min. Afterwards, 0.242 g Cu(NO_3_)_2_·3H_2_O (analytically pure) was added into the solution, and then the obtained dispersion was stirred for another 20 min. Subsequently, 10 mL sodium hydroxide solution (1 mol·L^−1^) was added dropwise with stirring. The mixture was then stirred at room temperature for two hours, then collected, washed with deionized water and alcohol, and dried. Finally, the mixture was calcined in a muffle furnace at 200 °C for two hours, to obtain the g-C_3_N_4_/CuO nanocomposite. CuO without g-C_3_N_4_ was also obtained using the same method under the same conditions.

### 2.3. Characterization

Unless otherwise indicated, g-C_3_N_4_/CuO nanocomposite containing 20 wt % CuO was used as a representative for characterization.

The crystalline phases of products were analyzed by X-ray diffraction (XRD) using a Rigaku Ultima-IV advanced X-ray diffractometer (Rigaku, Tokyo, Japan) with Cu Kα irradiation (λ = 0.15406 nm) within the range of 2θ = 10°–90°. The morphology and structure of the as-synthetized nanocomposite was observed by field emission scanning electron microscopy (FESEM, Zeiss MERLIN Compact, Jena, Germany) and transmission electron microscopy (TEM, Philips Tecnai 12, Amsterdam, The Netherlands). X-ray photoelectron spectra (XPS) were recorded on a PHI Quantera II X-ray photoelectron spectrometer (Chigasaki, Japan), using Al Kα radiation as the excitation source.

### 2.4. Catalytic Activity Measurement

For study of the catalytic performance of g-C_3_N_4_/CuO nanocomposite on the thermal decomposition of AP, AP powder mixed with g-C_3_N_4_, CuO, and g-C_3_N_4_/CuO were prepared.

For sample preparation for catalytic research, 0.01 g g-C_3_N_4_, CuO or g-C_3_N_4_/CuO was mixed with 0.49 g AP in ethanol, stirred for one hour, and then dried at 60 °C for one hour.

The resulting powder was determined by differential thermal analysis (DTA) (NETZSCH Instrument 404 PC, Selb, Germany) and thermal gravimetric analysis (TGA) (Beijing Hengjiu Instrument HTG-1, Beijing, China) at a heating rate of 10 °C min^−1^ (from 100–500 °C) under an N_2_ atmosphere.

## 3. Result and Discussion

### 3.1. Morphology and Textural Property

**Figure 1:**
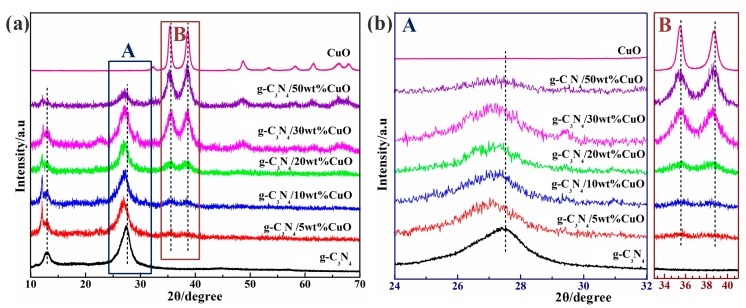
(**a**) X-ray diffraction (XRD) patterns of pure g-C_3_N_4_, CuO and g-C_3_N_4_/CuO nanocomposites with various contents of CuO; (**b**) Enlarged profiles of the selected area in Figure 1a.

The crystal structures of pure g-C_3_N_4_, CuO, and g-C_3_N_4_/CuO nanocomposite with various CuO contents were studied by XRD. As illustrated in Figure 1a, the g-C_3_N_4_ sample had a characteristic peak at 27.4° and 13.2°, which could be indexed by the hexagonal g-C_3_N_4_ (JCPDS87-1526) as the (002) and (100) diffraction plane peaks, corresponding to the interlayer stacking of aromatic segments with a distance of 0.326 nm, and the in-plane structural packing motif, respectively [32,33]. The diffraction peaks of the obtained CuO were indexed to the monoclinic phase of CuO (JCPDS05-0661) [34]. The diffraction peaks at 32.4°, 35.6°, 38.8°, 48.7°, 53.5°, 58.3°, 61.5°, 66.1°, 68.1°, 72.4°, 75.2° were observed (Figure 1a), corresponding to (1, 1, 0), (−1, 1, 1), (1, 1, 1), (−2, 0, 2), (0, 2, 0), (2, 0, 2), (−1, 1, 3), (3, 1, −1), (2, 2, 0), (3, 1, 1) and (2, 2, −2) [35]. The g-g-C_3_N_4_/CuO nanocomposite exhibited diffraction peaks corresponding to both g-C_3_N_4_ and CuO, reflecting the presence of two phases, and no other impurity peaks were observed. The enlarged profiles of the g-C_3_N_4_, CuO and all the g-C_3_N_4_/CuO nanocomposites are shown in Figure 1b. With an increased content of CuO in the g-C_3_N_4_/CuO nanocomposite, the characteristic peaks of g-C_3_N_4_ were gradually decreased, and the diffraction peaks of CuO were all slowly increased, indicating the successful synthesis of g-C_3_N_4_/CuO nanocomposite [36]. Nevertheless, the (002) reflection peak of the laminated g-C_3_N_4_ gradually shifted to a small angle direction along with an increased loading percentage of CuO nanorods on g-C_3_N_4_, which indicated that a certain number of CuO nanorods were grown in situ on the inter-lamination of g-C_3_N_4_, resulting in the increase of inter-layer spacing [37].

**Figure 2:**
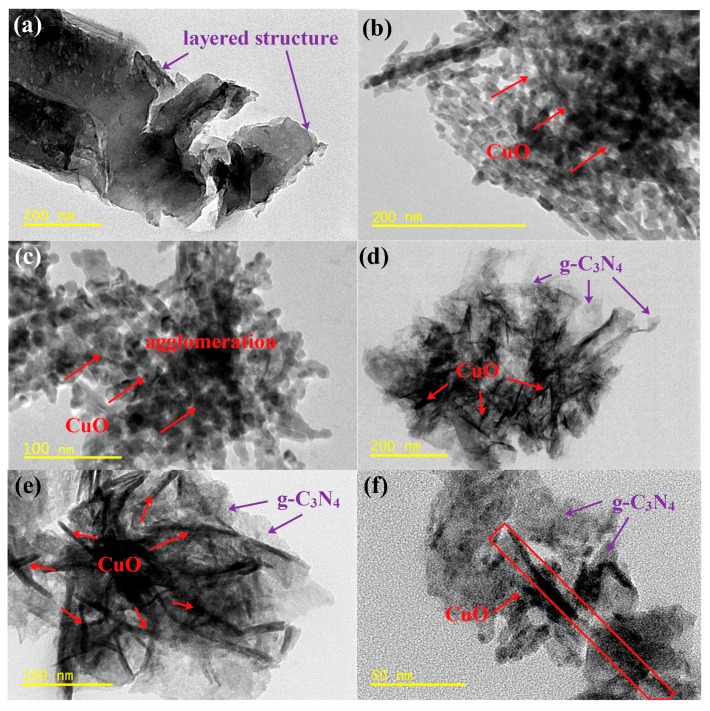
Transmission electron microscopy (TEM) images of (**a**) pure g-C_3_N_4_, (**b**,**c**) CuO, and (**d**–**f**) g-C_3_N_4_/20 wt % CuO nanocomposite.

The morphology of g-C_3_N_4_, CuO and g-C_3_N_4_/CuO nanocomposite (20 wt % CuO as a representative) was investigated by TEM and FESEM, and the TEM result is described in Figure 2. Figure 2a confirmed that g-C_3_N_4_ displayed a 2D laminated structure, which is similar to its graphite analogue, containing several stacking layers [38]. As shown in Figure 2b,c, pure CuO showed a dense agglomeration of rough nanoscale rods and spherical particles with an average diameter of 5–10 nm. However, the morphology of g-C_3_N_4_/CuO was obviously different from pure g-C_3_N_4_ and CuO. Figure 2d clearly demonstrated that CuO nanorods (labeled with red arrows) were well dispersed in g-C_3_N_4_. The light portion was g-C_3_N_4_, and the nanorod-shaped dark portion was CuO, which confirmed that CuO nanorods were directly grown on g-C_3_N_4_. Previous studies have indicated that g-C_3_N_4_ exhibits a strong ability to capture cations via an ion-dipole interaction between cations and lone pairs of electrons on nitrogen atoms of g-C_3_N_4_ [39,40]. Thus, after dispersing the g-C_3_N_4_ into the solution with cupric ions, the cupric ions could be captured and randomly dispersed into the framework of g-C_3_N_4_ (Scheme 1). In this case, upon the addition of hydroxide solution, the nano-Cu(OH)_2_ could be formed in situ because of the ion-dipole interaction, efficiently avoiding agglomeration. In addition, compared with the bulk g-C_3_N_4_ from Figure 2a, the color of g-C_3_N_4_ in g-C_3_N_4_/CuO nanocomposite (Figure 2d,e) was brighter, which indicated thinner lamellar thickness. These results demonstrated that the interlayer-formed CuO nanorods (synthesized by the calcination of as-prepared Cu(OH)_2_ at 200 °C) in return caused the partial exfoliation of the bulk g-C_3_N_4_, which was also supported by XRD results (Figure 1). Figure 2e,f show the TEM images with high magnification of the g-C_3_N_4_/CuO nanocomposite. We observed that the diameter of CuO nanorods ranged between 5–10 nm and the length was approximately 200–300 nm. As shown in Figure 2e, the overlapping phenomenon of CuO nanorods resulted from their simultaneous existence on the surface and inter-lamination of g-C_3_N_4_.

**Figure 3:**
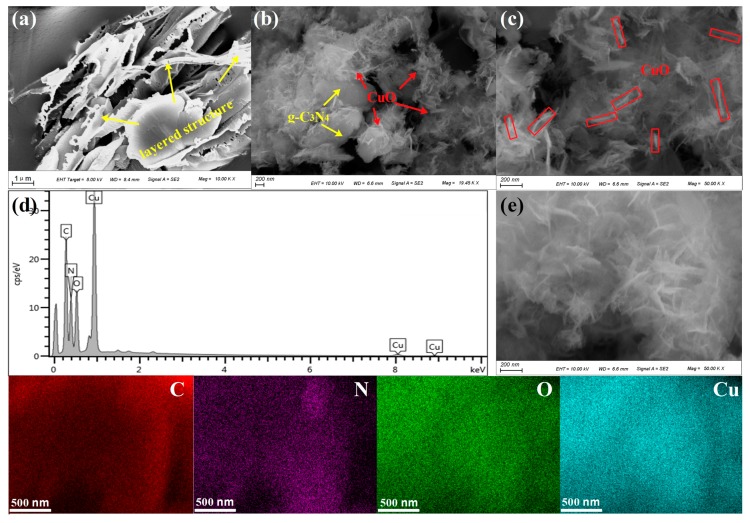
Field emission scanning electron microscopy (FESEM) images of (**a**) pure g-C_3_N_4_, (**b**,**c**) g-C_3_N_4_/CuO nanocomposite; (**d**) energy dispersive spectrometer (EDS) spectrum and (**e**) elemental mappings of the g-C_3_N_4_/20 wt % CuO nanocomposite.

Figure 3a further revealed that the pure g-C_3_N_4_ had loose laminated structures adhered between interlaminations [41]. From Figure 3b,c, CuO nanorods with a diameter of about 5–10 nm, and a length of 200–300 nm (labelled with red frame) were found to be dispersed on g-C_3_N_4_. We also employed energy dispersive spectrometer (EDS) to conduct elemental analysis on the nanocomposite, and characteristic peaks corresponding to C, N, Cu, and O were present in the EDS spectrum (Figure 3d). In order to further investigate the elemental distribution of the g-C_3_N_4_/CuO nanocomposite, the elemental mappings of C, N, Cu and O were also performed by EDS area scanning (Figure 3e). The maps of C, N, Cu and O were well defined, with sharp contrast. The maps of C and N showed C and N signals, respectively, indicating the definite existence of g-C_3_N_4_. The profile of Cu was similar to that of O, demonstrating the homogeneous distribution of CuO nanorods on g-C_3_N_4_. All these demonstrated the successful decorating of g-C_3_N_4_ with CuO nanorods. 

**Figure 4:**
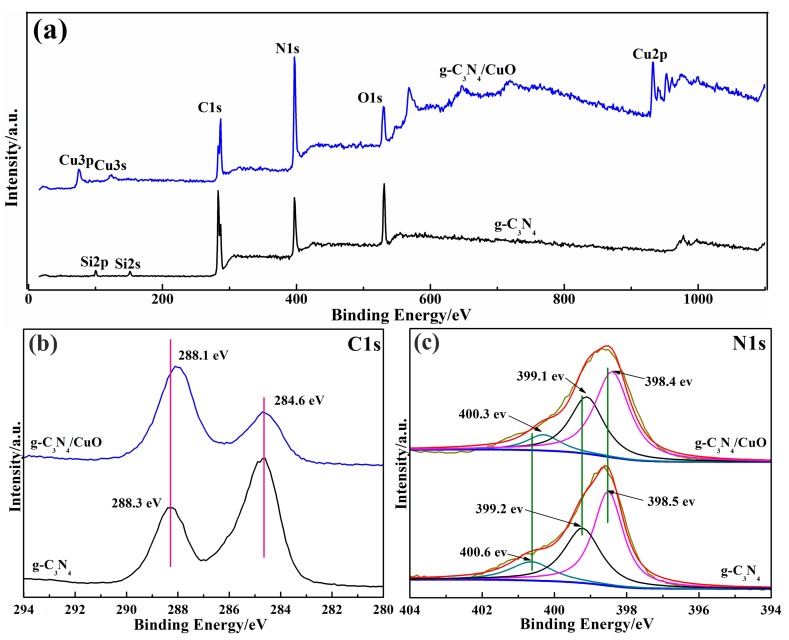
X-ray photoelectron spectra (XPS) of g-C_3_N_4_ and g-C_3_N_4_/20 wt % CuO nanocomposite: (**a**) full scan; (**b**) high resolution C1s; (**c**) high resolution N1s.

XPS was performed to further confirm the composition and the chemical states of the nanocomposite. The results obtained in the XPS analysis were corrected by referencing the C1s line to 284.6 eV. The full scan of the XPS spectra of g-C_3_N_4_ (Figure 4a) showed that characteristic peaks corresponding to C, N, O and Si elements were found. The existence of the O1s peak was due to the existence of O_2_ on the surface of the sample [42], and the existence of Si2p was attributed to the introduction of trace SiO_2_ during grinding. Meanwhile, characteristic peaks corresponding to C, N, O, and Cu were found in the full scan spectra of g-C_3_N_4_/CuO, and no other peaks could be assigned. All these demonstrated the successful preparation of the g-C_3_N_4_/CuO nanocomposite. Figure 4b,c are high-resolution XPS spectra of the C1s, and N1s. There were two peaks present in the C1s spectrum of g-C_3_N_4_, and the peak at 284.6 eV could be ascribed to the surface adventitious reference carbon, whereas the binding energy at 288.3 eV was corresponding to sp^2^-hybrid carbon of the N–C=N group [43]. The C1s spectrum of g-C_3_N_4_/CuO was similar to that of g-C_3_N_4_, while the blue shift (0.2 eV) of the binding energy at 288.1 eV indicated the interaction between g-C_3_N_4_ and CuO. As for the N1s high-resolution spectrum of g-C_3_N_4_, three binding energy peaks at 398.5, 399.2 and 400.6 eV could be assigned to the aromatic N(C=N–C), tertiary N(N–(C)_3_), and the N atom on C–N–H group, respectively [44,45]. The blue shift (0.1 eV) of the aromatic N(C=N–C) in the N1s spectrum of g-C_3_N_4_/CuO (Figure 4c) also supported the strong interaction between g-C_3_N_4_ and CuO.

### 3.2. Catalytic Activity of Nanocomposite Materials for Ammonium Perchlorate (AP) Decomposition

**Figure 5:**
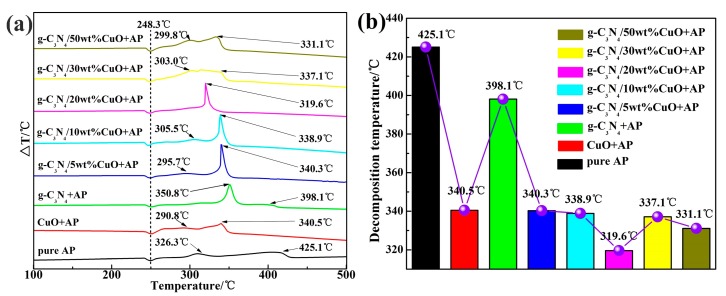
(**a**) Differential thermal analysis (DTA) curves of ammonium perchlorate (AP) in the absence and presence of a catalyst; (**b**) histogram of the corresponding high decomposition temperature.

**Figure 6:**
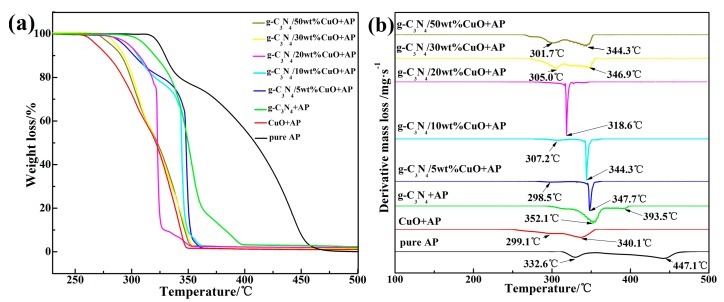
Thermal gravimetric analysis (TGA) (**a**) and derivative thermogravimetric analysis (DTG) (**b**) curves of ammonium perchlorate (AP) in the absence and presence of catalysts.

The thermal decomposition of AP was employed to evaluate the catalytic effect of g-C_3_N_4_/CuO nanocomposite. Specifically, AP in the absence and presence of 2 wt % pure g-C_3_N_4_, CuO, and g-C_3_N_4_/CuO (various content of CuO from 5 to 50 wt % versus g-C_3_N_4_/CuO), respectively, were utilized to investigate its thermal decomposition by differential thermal analysis (DTA) and thermal gravimetric analysis (TGA), and the results were illustrated in Figure 5 and Figure 6. The thermal decomposition process of AP in the absence of catalyst exhibited two exothermic peaks at 326.3 and 425.1 °C, corresponding to the low and high temperature decomposition stages [46], and one endothermic peak at 248.3 °C, which was the crystal transformation temperature of AP [47] (Figure 5). The addition of catalyst led to significant change in both high and low decomposition temperatures, while did not affect the crystal transformation temperature. When treated with 2 wt % CuO and 2 wt % g-C_3_N_4_ separately, the high decomposition temperature (HTD) of AP was decreased by 84.6 °C, and 27.0 °C, suggesting that pure CuO exhibited higher catalytic performance on thermal decomposition of AP than pure g-C_3_N_4_, which is in accordance with the literature [48]. Besides, with CuO content increased from 5 to 20 wt % in the g-C_3_N_4_/CuO nanocomposite, the HTD of AP decreased gradually. Specifically, upon the addition of 2 wt % g-C_3_N_4_/20 wt % CuO nanocomposite as a catalyst, the HTD of AP showed a maximum decrease of 105.5 °C, and only one peak corresponding to decomposition temperature was present in the spectrum, which was presumably due to the merging of high and low decomposition temperatures. In the case of the g-C_3_N_4_/CuO nanocomposite, CuO were all nano-sized and well dispersed throughout the micron-sized g-C_3_N_4_. Previous studies have indicated that active sites exposed on the surface of nanocomposite would significantly increase the enhancement well dispersed nano-component concentrations to some extent [49,50]. Thus, g-C_3_N_4_/20 wt % CuO exhibited higher catalytic performance on the thermal decomposition of AP than g-C_3_N_4_/10 wt % CuO and g-C_3_N_4_/5 wt % CuO, respectively. Meanwhile, on account of the partial agglomeration of CuO nanorods on g-C_3_N_4_ with the percentage of CuO being more than 20 wt % versus g-C_3_N_4_/CuO (g-C_3_N_4_/30 wt % CuO and g-C_3_N_4_/50 wt % CuO), the catalytic efficacy of the g-C_3_N_4_/CuO nanocomposite was decreased (Table 1); this was accompanied by the recurrence of two exothermic peaks corresponding to high and low decomposition temperatures, respectively.

The TG and DTG curves in Figure 6 showed that the decomposition process of AP has two platforms, indicating that there are two weight loss steps of AP from 100 to 500 °C [51]. It was also discovered that the initial and final weight-loss decomposition temperatures decreased in the presence of all independent catalysts (Figure 6a). In particular, with 2 wt % g-C_3_N_4_ and 2 wt % CuO, separately, both the two weight-loss decomposition temperatures of AP were decreased, and the decomposition process still exhibited two platforms corresponding to initial and final weight-loss decomposition. However, it should be noted that the addition of g-C_3_N_4_/CuO reduced the temperature span between the initial and final weight-loss decomposition temperatures, and the span disappeared with 20 wt % CuO (versus g-C_3_N_4_/CuO) added, resulting in a merge into one peak at 318.6 °C (maximum decrease by 128.5 °C) in the spectrum (Figure 6b), which is consistent with the observation from DTA. Meanwhile, the recurrence of two peaks corresponding to the initial and final weight-loss decomposition temperatures, when treated with g-C_3_N_4_/CuO containing more than 20 wt % CuO, implies that the catalytic efficacy of the g-C_3_N_4_/CuO nanocomposite decreased (Table 1). Overall, our observations indicated that g-C_3_N_4_ and CuO show synergistic catalytic effects on the thermal decomposition of AP.

The proposed thermal decomposition process of AP catalyzed by g-C_3_N_4_/CuO was also studied. The low temperature decomposition was a solid-gas heterogeneous reaction, which included a proton transfer to form NH_3_ and HClO_4_, the adsorption of NH_3_ and HClO_4_ in the porous structure, the decomposition of HClO_4_, and a subsequent reaction with NH_3_ [30]. The high temperature gas-phase decomposition reaction included the oxidation of NH_3_ by HClO_4_ in the gas phase [52].

Previous studies demonstrated that proton transfer was the most important process of the thermal decomposition of AP. Indeed, the as-prepared g-C_3_N_4_/CuO nanocomposite effectively promoted proton transfer. 

On one hand, Chen et al illustrated that the surface O^2−^ species of the CuO nanorod were likely to be proton traps which could react with NH_4_^+^ easily and then promote the process of proton transfer [53]. On the other hand, due to the unique surface structure of g-C_3_N_4_ that could be considered to be a Lewis base [54], HClO_4_ (generated from proton transfer reaction) could be absorbed on the surface of g-C_3_N_4_ via a Lewis acid-base interaction, leading to the separation of HClO_4_ from the pores of AP, and then resulting in a decrease of activation energy, thus accelerating the thermal decomposition of AP [55,56]. In summary, by combining the functions of g-C_3_N_4_ and CuO, the as-prepared g-C_3_N_4_/CuO nanocomposite would promote proton transfer effectively, and accelerating the thermal decomposition process of AP.

## 4. Conclusions

The novel nanocomposite g-C_3_N_4_/CuO, was synthesized through a facile precipitation method, and fully characterized using various methods. Specifically, on account of the ion-dipole interaction between cupric ions and long pair electrons on the nitrogen atoms of g-C_3_N_4_, CuO nanorods with diameter in the range of 5–10 nm and length of 200–300 nm were directly grown on g-C_3_N_4_. Moreover, g-C_3_N_4_/CuO greatly accelerated the thermal decomposition of AP. Upon addition of g-C_3_N_4_/CuO, the decomposition temperature of AP was decreased by up to 105.5 °C, and only one decomposition step was found, demonstrating synergetic catalytic activity of g-C_3_N_4_ and CuO. g-C_3_N_4_ was decorated with other promising metal oxides, which could broaden the application of g-C_3_N_4_. This potential application is currently being examined by our group.

## Figures and Tables

**Table 1:** High decomposition temperature and high weight-loss decomposition temperature of ammonium perchlorate (AP) in the absence and presence of different catalyst.

Sample	High Decomposition Temperature (°C)	High Weight-Loss Decomposition Temperature (°C)
Pure AP	425.1	447.1
CuO + AP	340.5	340.1
g-C_3_N_4_ + AP	398.1	393.5
g-C_3_N_4_/5 wt % CuO + AP	340.3	347.7
g-C_3_N_4_/10 wt % CuO + AP	338.9	344.3
g-C_3_N_4_/20 wt % CuO + AP	319.6	318.6
g-C_3_N_4_/30 wt % CuO + AP	337.1	346.3
g-C_3_N_4_/50 wt % CuO + AP	331.1	344.3
